# Seasonal Influenza Vaccination at a German University Hospital: Distinguishing Barriers Between Occupational Groups

**DOI:** 10.3389/fmed.2022.873231

**Published:** 2022-05-27

**Authors:** Martin Peschke, Stefan Hagel, Norman Rose, Mathias W. Pletz, Andrea Steiner

**Affiliations:** ^1^Occupational Health Service, Jena University Hospital, Friedrich-Schiller-University Jena, Jena, Germany; ^2^Institute for Infectious Diseases and Infection Control, Jena University Hospital, Friedrich-Schiller-University Jena, Jena, Germany

**Keywords:** influenza vaccination, health care personnel, occupation, hospital, vaccination uptake

## Abstract

The annual influenza vaccination has been officially recommended for medical staff in Germany since 1988. Nevertheless, the vaccination rate among medical staff is still low. The present study deals with the influenza vaccination rate of staff at a German University hospital over time as well as with the reasons that led to a positive vaccination decision and the barriers to acceptance of vaccination. For this purpose, the staff members received questionnaires in which they were asked about influenza vaccination and the reasons for or against vaccination. In addition, the questionnaire contains information on gender, age group, occupational group and presence of a chronic co-morbidity. Logistic regression analysis was used to investigate which of these predictors most strongly influenced the vaccination decision. It was shown that the reasons for or against vaccination differ significantly between the occupational groups and that the occupational group affiliation has the greatest influence on the vaccination decision in the comparison of the investigated predictors. In order to achieve a positive influence on vaccination acceptance, future measures should focus on increasing confidence in vaccination and on increasing the perception of risk from influenza illness. The findings may contribute to future targeted strategies to increase vaccination rates and suggest occupational group-specific interventions.

## Introduction

Seasonal influenza is largely responsible for mortality, morbidity and sick leave (1, 2). Annual influenza vaccination is the most effective method of controlling influenza and is recommended for healthcare workers and at-risk-groups but is also useful for the general population (3, 4). Nevertheless, the influenza vaccination rate among medical personnel is still low and also in Germany, at 40%, we are far from the WHO goal of 75% (5, 6). Recommendations and programmes to increase vaccination rates have not led to the desired success (7, 8). In order to be able to increase vaccination adequately, an analysis of the reasons for psychological barriers to the uptake of vaccination is necessary (9). This allows subsequently to reduce specific prejudices, dispel reservations and increase confidence in vaccination (10). The current study was conducted to find out, whether the reasons that led to non-uptake of vaccination differed between the occupational groups of a large German University hospital and which of the predictors studied had the greatest influence on the decision to vaccinate. At our hospital, seasonal influenza vaccination has been recommended and offered free of charge for more than 10 years. However, especially in the nursing sector, i.e., in an area with the highest patient contact, vaccination acceptance is still unsatisfactorily low. For this study, questionnaires from employees were analyzed asking for influenza vaccination status for two consecutive seasons (2018/2019 and 2019/2020) and their intention to get or get not vaccinated in the 2020/2021 season as well as reasons for or against vaccination and personal information on gender, age group and occupational group.

## Methods

### Participants and Procedure

The University Hospital Jena (UKJ) is a maximum care hospital with 1,396 beds and approx. Six Thousand employees, it is the largest hospital in the State of Thuringia in Germany. We handed out a questionnaire as paper to employees between September 2019 and March 2020 as part of the regular occupational health consultation hours, which was to be filled in anonymously. In Germany, for healthcare workers it is mandatory to regularly attend occupational health consultations. This applies regardless of age, gender or specific occupational group. There are no periods that apply exclusively to a specific group. This ensured that the survey was conducted on a random basis. Five-hundred-sixty-eight employees from various occupational groups took part in the survey. We did not enroll recently hired employees, who came to the first consultation before starting work or if the start of work was <6 months ago and could therefore not be familiar with the UKJ vaccination policies.

### The Questionnaire

The questionnaire (Supplementary Material) consisted of 11 parts. In addition to age, gender, occupational group and presence of a chronic illness, the questionnaire asked about influenza vaccination in the previous season (18/19) the current season (19/20) and the intention to get or get not vaccinated for the incoming season (20/21). In addition, it asked who had carried out the previous vaccination and whether the employees were aware of the offer of free influenza vaccination. To describe the personal motivation for vaccination, the participants could choose from five pre-formulated answer options, for the motivation against vaccination from 14 options. In each case, a free field to describe not-listed own reasons was available. Multiple answers were possible. The aim was to investigate whether there are differences in vaccination acceptance between the individual occupational groups and if age, gender or the presence of a medically diagnosed chronic disease had an additional impact.

### Data Analysis

The statistical analyses of changes in the vaccination rate among hospital staff over the three influenza seasons (2018/19, 2019/20, and 2020/21) were based on two-level regression models, with the timepoints as level-1 and persons as level-2 units. In order to test the null hypothesis of no change in the vaccination rate over time, we used a likelihood ratio test of two nested models: (a) The random-intercept model with two dummy variables for the influenza seasons 2019/20 and 2020/21, and (b) the random-intercept-only model (i.e., the null model). The likelihood ratio test was conducted for all occupational groups.

Potential differences in the vaccination coverage between occupational groups, age groups, gender and persons with and without a chronic disease were tested by means of χ^2^-tests with CramersV as the effect size measure. The influenza vaccination status in 2019/20 was the dependent variable. The reason is that influenza season 2019/20 coincides with the time when the questionnaire was answered by the medical staff. In addition, the influenza season 2019/20 was the last one that can be assumed not to be affected by the Covid-19 pandemic. Accordingly, attitudes and decision-making regarding the influenza vaccination were not influenced by the pandemic.

In the sub-sample of vaccinated individuals, the differences in the reported reasons for the influenza vaccination between different occupational groups were tested using χ^2^-tests with *p*-values adjusted for multiple tests according to Holm (11). CramersV was chosen as the effect size measure. The same statistical procedure was used in the sub-sample of unvaccinated individuals for the differences in the reported reasons against the influenza vaccination. In the case of zero cells in the contingency tables, the *p*-value of the χ^2^-tests were obtained by non-parametric bootstrap with 10.000 bootstrap samples. A significance level of α = 0.05 was chosen for statistical hypothesis tests. All statistical analyses were conducted using the free statistical software R and the R package DescTools (12, 13). Percentages of vaccination uptake and proportions are reported with 95% Wilson score confidence interval.

## Results

The study includes a total of 568 participants. We distributed 899 questionnaires, the response rate was 63.2%. The majority were female (369; 69.7%), of whom 16.9% reported a chronic co-morbidity. One hundred and seventy-two respondents (30.3%) were male, of whom 14.5% reported a chronic co-morbidity. Two-hundred-eight were students, 123 nurses, 80 physicians, 44 administrative staff, 44 laboratory staff (microbiology, clinical chemistry), 45 medical-technical staff (for example medical radiation technologists) and 24 who classified themselves as “others”. Five employees were under 18 years of age, 162 18–24, 157 25–34, 130 35–44, 88 45–54 and 25 older than 55. We found that the influenza vaccination rate among the occupational groups increased statistically significant within each occupational group during the period studied (*p* < 0.001 in all groups, see Table 1). In the following description of the results we focus on the influenza season 2019/20. We found the highest vaccination rate among physicians with 66% and the lowest among nursing staff with 31% and this difference was statistically significant (*p* = 0.001). The vaccination rate among men was also significantly higher (59.9%) than among women (46.5%, *p* = 0.004). Only small differences were found between the six age groups (< 18 years = 80.0%, 18–24 years = 50.6%, 25–34 years = 47.8%, 35–44 years = 50.8%, 45–54 years = 48.9%, ≥ 55 years = 42.3%). Surprisingly, medical staff with and without a chronical illness did not differ in their vaccination rates (not chronically ill = 49.5%, chronically ill = 49.4%). As shown in Table 2, the occupational group has the strongest relationship with vaccination rate and is the most decisive predictor. In addition, we found significant differences between the different occupational groups and the selection of reasons for vaccination (see Table 3). The reason with the weakest relationship to the occupational group was the protection of one's own health (Cramer'sV = 0.205). This was found to be the most important reason for vaccination for every occupational group, and thus even more important than protecting the patients. Interestingly, the occupational groups also differed significantly on the question of whether the recommendation of vaccination played a role (*p* < 0.001). Here, a medium effect size was found (Cramer'sV 0.381). Thus, among the medical-technical staff, official vaccination recommendation accounted for 70% of the reasons to get vaccinated, whereas in the “others” group it accounted for 0%. The occupational groups also differed substantially in reasons against vaccination (see Table 4). Whereas, general reluctance to vaccinate, the trust in herd immunity and not having taken up the vaccination because someone advised against it were rarely found reasons in the different groups, physicians mainly stated that the vaccination was not effective or not effective enough and the fear of the vaccination as a flu trigger played the major role among the nursing staff. Students (100%) expressed the opinion that they would not fall ill with influenza, administrative staff members considered themselves not to be at increased risk for infection and the laboratory personnel expressed to generally distrust vaccinations and have no fear of falling ill with influenza. The medical-technical staff rejected the vaccination mainly because of the poor benefit/risk ratio and the “others” showed a very heterogeneous picture of reasons for rejection. The low vaccination rate among nursing staff is mainly due to a lack of confidence in vaccination and reveals typical prejudices, such as that influenza vaccination causes influenza illness. Tables 3, 4 show the results of the analysis for differences between the occupational groups regarding reasons for and against vaccination.

**Table 1:** Demographics and absolute numbers and percentages of vaccinated individuals in the influenza seasons 2018/19, 2019/20, and 2020/21.

				**Vaccinated**		
		**Female**	**Chronically ill**	**Season 2018/19**	**Season 2019/20**	**Season 2010/21**
**Occupational group**	***N* (%)**	***N* (%)**	***N* (%)**	**N (% [95%-CI])**	**N (% [95%-CI])**	**N (% [95%–CI])**
Physicians	80 (14,1%)	45 (56,2%)	16 (20,0%)	40 (50,0% [39,3, 60,7])	53 (66,2% [55,4, 75,7])	68 (85,0% [75,6, 91,2])
Nursing staff	123 (21,7%)	101 (82,1%)	24 (19,5%)	22 (17,9% [12,1, 25,6])	39 (31,7% [24,1, 40,4])	73 (59,3% [50,5, 67,6])
Students	208 (36,6%)	137 (65,9%)	13 (6,2%)	60 (28,8% [23,1, 35,3])	106 (51,0% [44,2, 57,7])	125 (60,1% [53,3, 66,5])
Administration	44 (7,7%)	27 (61,4%)	9 (20,5%)	20 (45,5% [31,7, 59,9])	29 (65,9% [51,1, 78,1])	33 (75,0% [60,6, 85,4])
Laboratory staff	44 (7,7%)	36 (81,8%)	10 (22,7%)	12 (27,3% [16,3, 41,8])	16 (36,4% [23,8, 51,1])	24 (54,5% [40,1, 68,3])
Medical-technical	45 (7,9%)	39 (86,7%)	10 (22,2%)	22 (48,9% [35,0, 63,0])	30 (66,7% [52,1, 78,6])	37 (82,2% [68,7, 90,7])
Others	24 (4,2%)	11 (45,8%)	7 (29,2%)	9 (37,5% [21,2, 57,3])	14 (58,3% [38,8, 75,5])	19 (79,2% [59,5, 90,8])
All	568 (100.0%)	396 (69.7%)	89 (15.7%)	185 (32.6% [28.8, 36.5])	287 (50.5% [46.4, 54.6])	379 (66.7% [62.7, 70.5])

**Table 2:** χ^2^-Test and effect size (Cramer's V) of the stochastic relationship between predictors and vaccination decision in the influenza season 2019/20.

**Predictor**	**χ^2^**	**Df**	**P-value**	**Cramer's V**
Occupational group	38,325	6	0,000	0,260 [0,156, 0,327]
Age	2,765	5	0,736	0,070 [0,000, 0,111]
Gender	8,110	1	0,004	0,123 [0,041, 0,206]
Presence of chronic disease	0,000	1	1,000	0,000 [0,000, 0,094]

**Table 3:** Relative frequencies, χ^2^-Test and effect size (Cramer's V) of the stochastic relationship between different reasons for vaccination and the occupational group.

	**Occupational group**									
**Reasons in favor of vaccination**	**Physicians**	**Nursing staff **	**Students **	**Administrative staff **	**Laboratory staff**	**Medical-technical staff**	**Others**	**χ^2^**	**Cramer's V**	**All groups**
Protection your own health	72.9% [61.5, 81.9]	52.1% [40.8, 63.1]	51.5% [43.0, 60.0]	73.5% [56.9, 85.4]	60.0% [40.7, 76.6]	73.0% [57.0, 84.6]	68.4% [46.0, 84.6]	16.376*	0.205 [0.047, 0.278]	60.8% [55.9, 65.6]
Patient protection	64.3% [52.6, 74.5]	41.1% [30.5, 52.6]	14.6% [9.6, 21.7]	0.0% [0.0, 10.2]	8.0% [2.2, 25.0]	59.5% [43.5, 73.7]	21.1% [8.5, 43.3]	91.650**	0.486 [0.371, 0.573]	31.4% [27.0, 36.2]
Protection your personal environment	57.1% [45.5, 68.1]	31.5% [22.0, 42.9]	41.5% [33.4, 50.1]	58.8% [42.2, 73.6]	48.0% [30.0, 66.5]	0.0% [0.0, 9.4]	52.6% [31.7, 72.7]	42.029**	0.329 [0.205, 0.411]	41.0% [36.2, 45.9]
Vaccination recommendation	11.4% [5.9, 21.0]	21.9% [14.0, 32.7]	24.6% [18.0, 32.7]	23.5% [12.4, 40.0]	16.0% [6.4, 34.7]	70.3% [54.2, 82.5]	0.0% [0.0, 16.8]	56.203**	0.381 [0.261, 0.465]	24.2% [20.2, 28.7]
Protection against loss of working hours	4.3% [1.5, 11.9]	9.6% [4.7, 18.5]	0.0% [0.0, 2.9]	0.0% [0.0, 10.2]	0.0% [0.0, 13.3]	10.8% [4.3, 24.7]	5.3% [0.9, 24.6]	18.969*	0.221 [0.073, 0.295]	3.9% [2.4, 6.3]

*p < 0.05,

**p < 0.001.

*In the case of zero cells the p-value was obtained by non-parametric bootstrap with 10000 bootstrap samples. p-values of χ^2^-tests are adjusted for multiple tests according to Holm*.

**Table 4:** Relative frequencies, χ^2^-Test, and effect size (Cramer's V) of the stochastic relationship between different reasons against vaccination and the occupational group.

	**Occupational group**									
**Reasons against vaccination**	**Physicians**	**Nursing staff**	**Students**	**Administrative staff**	**Laboratory staff**	**Medical-technical Staff**	**Others**	**χ^2^**	**Cramer's V**	**All groups**
Organizational reasons	0.0% [0.0, 27.8]	0.0% [0.0, 7.1]	19.2% [12.0, 29.3]	20.0% [5.7, 51.0]	21.1% [8.5, 43.3]	62.5% [30.6, 86.3]	20.0% [3.6, 62.4]	26.680*	0.385 [0.188, 0.499]	15.0% [10.5, 20.9]
I have forgotten it	0.0% [0.0, 27.8]	4.0% [1.1, 13.5]	30.8% [21.6, 41.7]	20.0% [5.7, 51.0]	0.0% [0.0, 16.8]	0.0% [0.0, 32.4]	40.0% [11.8, 76.9]	26.385**	0.383 [0.186, 0.497]	16.7% [11.9, 22.8]
I was advised against	0.0% [0.0, 27.8]	0.0% [0.0, 7.1]	15.4% [9.0, 25.0]	0.0% [0.0, 27.8]	0.0% [0.0, 16.8]	0.0% [0.0, 32.4]	0.0% [0.0, 43.4]	16.813**	0.306 [0.076, 0.412]	6.7% [3.9, 11.3]
I am generally against vaccinations	0.0% [0.0, 27.8]	6.0% [2.1, 16.2]	15.4% [9.0, 25.0]	0.0% [0.0, 27.8]	0.0% [0.0, 16.8]	0.0% [0.0, 32.4]	0.0% [0.0, 43.4]	10.161**	0.238 [0.000, 0.334]	8.3% [5.1, 13.3]
I do not trust the official recommendations	0.0% [0.0, 27.8]	12.0% [5.6, 23.8]	0.0% [0.0, 4.7]	50.0% [23.7, 76.3]	36.8% [19.1, 59.0]	0.0% [0.0, 32.4]	40.0% [11.8, 76.9]	44.314*	0.496 [0.315, 0.617]	11.1% [7.3, 16.5]
I am not aware that I should be vaccinated	30.0% [10.8, 60.3]	4.0% [1.1, 13.5]	32.1% [22.7, 43.0]	0.0% [0.0, 27.8]	0.0% [0.0, 16.8]	0.0% [0.0, 32.4]	0.0% [0.0, 43.4]	28.748*	0.400 [0.206, 0.515]	16.7% [11.9, 22.8]
I am unlikely to get flu	0.0% [0.0, 27.8]	0.0% [0.0, 7.1]	100.0% [95.3, 100.0]	0.0% [0.0, 27.8]	52.6% [31.7, 72.7]	50.0% [21.5, 78.5]	40.0% [11.8, 76.9]	148.190**	0.907 [0.744, 1.000]	52.2% [45.0, 59.4]
Vaccination is ineffective	100.0% [72.2, 100.0]	0.0% [0.0, 7.1]	0.0% [0.0, 4.7]	0.0% [0.0, 27.8]	21.1% [8.5, 43.3]	0.0% [0.0, 32.4]	0.0% [0.0, 43.4]	135.974**	0.869 [0.705, 1.000]	7.8% [4.7, 12.6]
I am afraid of side effects	0.0% [0.0, 27.8]	18.0% [9.8, 30.8]	30.8% [21.6, 41.7]	70.0% [39.7, 89.2]	0.0% [0.0, 16.8]	0.0% [0.0, 32.4]	40.0% [11.8, 76.9]	27.417**	0.390 [0.195, 0.505]	23.3% [17.8, 30.0]
Vaccination can trigger a flu illness	0.0% [0.0, 27.8]	60.0% [46.2, 72.4]	10.3% [5.3, 19.0]	0.0% [0.0, 27.8]	26.3% [11.8, 48.8]	50.0% [21.5, 78.5]	0.0% [0.0, 43.4]	51.127*	0.533 [0.355, 0.656]	26.1% [20.2, 33.0]
I do not pose a risk to patients	0.0% [0.0, 27.8]	6.0% [2.1, 16.2]	11.5% [6.2, 20.5]	100.0% [72.2, 100.0]	15.8% [5.5, 37.6]	0.0% [0.0, 32.4]	20.0% [3.6, 62.4]	65.841*	0.605 [0.432, 0.730]	14.4% [10.1, 20.3]
Vaccination carries more risks than benefits	100.0% [72.2, 100.0]	44.0% [31.2, 57.7]	11.5% [6.2, 20.5]	0.0% [0.0, 27.8]	26.3% [11.8, 48.8]	62.5% [30.6, 86.3]	20.0% [3.6, 62.4]	50.318**	0.529 [0.350, 0.651]	28.9% [22.8, 35.9]
It is enough if co-workers are vaccinated	0.0% [0.0, 27.8]	0.0% [0.0, 7.1]	0.0% [0.0, 4.7]	0.0% [0.0, 27.8]	0.0% [0.0, 16.8]	0.0% [0.0, 32.4]	20.0% [3.6, 62.4]	35.196**	0.442 [0.255, 0.560]	0.6% [0.1, 3.1]
I did not tolerate the vaccination	30.0% [10.8, 60.3]	0.0% [0.0, 7.1]	0.0% [0.0, 4.7]	0.0% [0.0, 27.8]	0.0% [0.0, 16.8]	0.0% [0.0, 32.4]	0.0% [0.0, 43.4]	51.864**	0.537 [0.359, 0.660]	1.7% [0.6, 4.8]

*p < 0.05,

**p < 0.001.

*In the case of zero cells the p-value was obtained by non-parametric bootstrap with 10000 bootstrap samples. p-values of χ^2^-tests are adjusted for multiple tests according to Holm*.

## Discussion

In this study, membership of an occupational group was the strongest influencing factor regarding the decision to get or get not vaccinated and the related underlying motivation. A significantly higher vaccination rate was found among physicians compared to other occupational groups. A finding that is in line with the literature (14–17). In general, our study found a higher vaccination rate compared to reports from other University hospitals (18–21). Our results provide detailed insights into the motivations of non-vaccinated staff and identifies barriers that need to be addressed in respective campaigns. Because the occupational group is the most decisive factor for the decision to get vaccinated, specific campaigns and strategies should be tailored to the needs of the individual occupational groups. In nursing staff, the unwarranted fear of contracting influenza through vaccination, the fear of side effects or the belief in a poor benefit/risk ratio dominated. A certain general vaccination skepticism was also detected. This could be addressed with a more comprehensive information program in which immunisations are a recurring topic. Physicians considered the vaccination to be not effective or not sufficiently effective but in contrast, fear of side effects or the belief in vaccination as a disease trigger did not play a role. Across all occupational groups, there was insufficient risk awareness of the disease. This seems to be a common issue, where educational programs need to start in order to draw attention to the dangers and possible consequences of a severe influenza illness and to raise awareness of the risk, especially for patients, but also for co-workers. The fact that the vaccination rate is currently so much higher among physicians than among nursing staff could indicate that the previous campaigns were tailored more to doctors than to address the barriers of nursing staff. At our hospital, the focus has so far been on breaking down logistic barriers in order to make access to vaccination as low-threshold as possible for employees. The strategy seems to have been successful in that organizational reasons such as “I didn't make it” do not play a relevant role across all occupational groups. Another strategy to further increase vaccination rates would be compulsory vaccination. A mandatory influenza vaccination introduced in the USA led to vaccination rates of over 90% (22). In Europe, and especially in Germany, however, a mandatory vaccination will probably fail due to general non-acceptance. In contrast, the implementation of specific measures to raise vaccination rates voluntarily by increasing confidence in the vaccine and awareness for the disease tailored to individual occupational groups to increase the influenza vaccination rate on a voluntary basis seems to be feasible. The main task here is to close gaps in knowledge and to correct existing misconceptions. This could be obtained through intensive information campaigns with flyers, videos on the intranet or special information events (23, 24). Occupational health consultations should also be used to provide comprehensive information about vaccination and disease. Specifically mandatory consultation by trained occupational physicians could be implemented for the unvaccinated. If convinced, the employees could also get the vaccination in this setting. Through this, it seems possible to both remove organizational barriers and address individual concerns and knowledge gaps, independent of occupational group.

### Limitations

The vaccination rate of the employees is based on self-reporting by the respondents on the basis of the questionnaire. It was not possible to check whether vaccination was actually carried out in this setting. The data for the 20/21 season reflects the intention of employees to be vaccinated against influenza in that season.

## Conclusions

The study provides an overview of the different levels of influenza vaccination coverage in different occupational groups. Occupational-group specific barriers and motivations for non-uptake of vaccination could be identified. It seems that these barriers have not yet been sufficiently addressed in an approach tailored to the specific occupational group. In the future, occupational group-specific strategies should be implemented to further increase the influenza vaccination rate especially in the area of nursing staff.

## Data Availability Statement

The raw data supporting the conclusions of this article will be made available by the authors, without undue reservation.

## Ethics Statement

The Ethics Committee of the Jena University Hospital approved the study. Written informed consent from the patients/participants was not required to participate in this study in accordance with the national legislation and the institutional requirements.

## Author Contributions

MWP, AS, MP, and SH: conceptualization. NR and MP: data analysis. MP: wrote the preliminary manuscript. MWP, AS, and MP: reviewed and revised the manuscript. All authors contributed to manuscript revision, read, and approved the submitted version.

## Conflict of Interest

The authors declare that the research was conducted in the absence of any commercial or financial relationships that could be construed as a potential conflict of interest.

## Publisher's Note

All claims expressed in this article are solely those of the authors and do not necessarily represent those of their affiliated organizations, or those of the publisher, the editors and the reviewers. Any product that may be evaluated in this article, or claim that may be made by its manufacturer, is not guaranteed or endorsed by the publisher.

## References

[1] Zaffina S Gilardi F Rizzo C Sannino S Brugaletta R Santoro A. Seasonal influenza vaccination and absenteeism in health-care workers in two subsequent influenza seasons (2016/17 and 2017/18) in an Italian pediatric hospital. Expert Rev Vaccines. (2019) 18:411–8. 10.1080/14760584.2019.1586541 30919703

[2] Ahmed F Lindley MC Allred N Weinbaum CM Grohskopf L. Effect of influenza vaccination of healthcare personnel on morbidity and mortality among patients. Systematic review and grading of evidence Clin Infect Dis. (2014) 58:50–7. 10.1093/cid/cit580 24046301

[3] Nicholson KG Snacken R Palache AM. Influenza immunization policies in Europe and the United States. Vaccine. (1995) 13:365–9. 10.1016/0264-410X(95)98258-C 7793132

[4] Simeonsson K Moore Z. Prevention and control of influenza: no easy task. N C Med J. (2013) 74:425–33. 10.18043/ncm.74.5.425 24165775

[5] Dini G Toletone A Sticchi L Orsi A Bragazzi NL Durando P. Influenza vaccination in healthcare workers: A comprehensive critical appraisal of the literature. Hum Vaccin Immunother. (2018) 14:772–89. 10.1080/21645515.2017.1348442 28787234 PMC5861785

[6] Jörgensen P Mereckiene J Cotter S Johansen K Tsolova S Brown C. How close are countries of the WHO European Region to achieving the goal of vaccinating 75% of key risk groups against influenza? Results from national surveys on seasonal influenza vaccination programmes, 2008/2009 to 2014/2015. Vaccine. (2018) 36:442–52. 10.1016/j.vaccine.2017.12.019 29287683 PMC5777640

[7] Llupià A Garcia-Basteiro AL Olivé V Costas L Rios J Quesada S. New interventions to increase influenza vaccination rates in health care workers. Am J Infect Control. (2010) 38:476–81. 10.1016/j.ajic.2010.01.013 20421140

[8] Mereckiene J Cotter S Nicoll A Lopalco P Noori T Weber J. Seasonal influenza immunisation in Europe. overview of recommendations and vaccination coverage for three seasons: prepandemic (2008/09), pandemic (2009/10) and post-pandemic (2010/11). Euro surveill. (2014) 19:20780. 10.2807/1560-7917.ES2014.19.16.20780 24786262

[9] Schmid P Rauber D Betsch C Lidolt G Denker M-L. Barriers of influenza vaccination intention and behavior: a systematic review of influenza vaccine hesitancy, 2005-2016. PLoS ONE. (2017) 12:e0170550. 10.1371/journal.pone.0170550 28125629 PMC5268454

[10] Kok G van Essen GA Wicker S Llupia A Mena G Correia R. Planning for influenza vaccination in health care workers: an intervention mapping approach. Vaccine. (2011) 29:8512–9. 10.1016/j.vaccine.2011.09.008 21939722

[11] Holm S. A simple sequentially rejective multiple test procedure. Scandinavian J Statistics. (1979) 6:65–70. Available online at: https://www.jstor.org/

[12] R Core Team. R A Language and Environment for Statistical Computing. Vienna: R Foundation for Statistical Computing (2020).

[13] Andri S Aho K Alfons A Anderegg N Aragon T Arachchige C. DescTools: Tools for Descriptive Statistics. R package version 0.99.44 (2021). Available online at: https://cran.r-project.org/package=DescTools

[14] Wilde JA McMillan JA Serwint J Butta J O'Riordan MA Steinhoff MC. Effectiveness of influenza vaccine in health care professionals: a randomized trial. JAMA. (1999) 281:908–13. 10.1001/jama.281.10.908 10078487

[15] Durovic A Widmer AF Dangel M Ulrich A Battegay M Tschudin-Sutter S. Low rates of influenza vaccination uptake among healthcare workers: Distinguishing barriers between occupational groups. Am J Infect Control. (2000) 48:1139–43. 10.1016/j.ajic.2020.02.004 32199740

[16] Nichol KL Hauge M. Influenza vaccination of healthcare workers. Infect Control Hosp Epidemiol. (1997) 18:189–94. 10.1086/647585 9090547

[17] Elias C Fournier A Vasiliu A Beix N Demillac R Tillaut H. Seasonal influenza vaccination coverage and its determinants among nursing homes personnel in western France. BMC Public Health. (2017) 17:634. 10.1186/s12889-017-4556-5 28687075 PMC5501011

[18] Keske S Mutters NT Tsioutis C Ergönül Ö. EUCIC influenza vaccination service team. Influenza vaccination among infection control teams: a ECIC survey prior to COVID-19 pandemic. Vaccine. (2020) 38:8357–61. 33183855 10.1016/j.vaccine.2020.11.003

[19] Squeri R Riso R Facciola A Genovese C Palamara M A R Ceccio C. Management of two influenza vaccination campaigns in health care workers of a University hospital in the south Italy. Ann Ig. (2017) 29:223–31. 10.7416/ai.2017.2150 28383614

[20] Giese C Mereckiene J Danis K O'Donnell J O'Flanagan D Cotter S. Low vaccination coverage for seasonal influenza and pneumococcal disease among adults at-risk and health care workers in Ireland, 2013: the key role of GPs in recommending. Vaccine. (2016) 34:3657–3662. 10.1016/j.vaccine.2016.05.028 27255466

[21] European Centre for Disease Prevention and Control. Seasonal influenza vaccination in Europe. Vaccination Recommendations and Coverage Rates in the EU Member States for Eight Influenza Seasons: 2007-2008 to 2014-2015. Stockholm: ECDC (2017)

[22] Maltezou HC Theodoridou K Ledda C Rapisarda V Theodoridou M. Vaccination of healthcare workers: is mandatory vaccination needed? Expert Rev Vaccines. (2019) 18:5–13. 10.1080/14760584.2019.1552141 30501454

[23] Hollmeyer H Hayden F Mounts A Buchholz U. Review: interventions to increase influenza vaccination among healthcare workers in hospitals. Influenza Other Respi Viruses. (2013) 7:604–21. 10.1111/irv.12002 22984794 PMC5781006

[24] To KW Lai A Lee KC Koh D Lee SS. Increasing the coverage of influenza vaccination in healthcare workers: review of challenges and solutions. J Hosp Infect. (2016)94:133–42. 27546456 10.1016/j.jhin.2016.07.003

