# Supplementary material for: Seasonal Influenza Vaccination at a German University Hospital: Distinguishing Barriers Between Occupational Groups
Source: Front Med (Lausanne). 2022 May 27;9:873231. doi: 10.3389/fmed.2022.873231 (PMC9184714; doi:10.3389/fmed.2022.873231)
Supplement: Supplementary file 1 [file Data_Sheet_1.docx]

1. Are you  male or  female?

2. How old are you?
 under 18 years

18 – 24 years
 25 – 34 years
 35 – 44 years
 45 – 54 years
 55 years and older

3. Do you have a medically diagnosed chronic disease?

Yes Neo

4. Which occupational group do you belong to?
 Physician
 Nursing staff
 Administration staff
 Student
 medical-technical staff
 Laboratory staff
 Others

5. Why did you get vaccinated? *(Multiple answers possible)*

To protect my own health
 In order to protect the health of the patients
 To protect my personal environment (family and friends)
 Because I was recommended the vaccination
 Because I can't be absent from work
 Other reasons: _____________

_____________________________________

6. Who carried out vaccination?
 General practitioner
 Occupational physician
 Myself
 Someone else

7. Why didn’t you get vaccinated? *(Multiple answers possible)*

I could not make it for organisational reasons

I have forgotten it
 I did not tolerate the vaccination
 I was advised against
 I am generally against vaccinations
 I do not trust the official recommendations
 I am not aware that I should be vaccinated
 I am unlikely to get flu
 The vaccination is ineffective
 I am afraid of side effects
 The flu vaccination can trigger a flu illness
 I do not pose a risk to patients
 It's enough if most of my co-workers are vaccinated against influenza
 When I weigh up the risks and benefits, vaccination comes off badly
 Other reasons: _____________

_____________________________________

8. Was the flu vaccination offered at your hospital during the last season, i.e. between september 2018 and march 2019 inclusive?

Yes  No  I do not know

9. Did you get vaccinated against flu last season?

Yes  No  I do not know

10. Are you planning to get vaccinated against influenza in the current season or have you already been vaccinated?

Yes  No  I do not know

11. Do you plan to get vaccinated against influenza in the coming season?

Yes  No  I do not know
